# O9-2 Participants' and leaders' experiences of a family-based health promotion programme: A Healthy Generation

**DOI:** 10.1093/eurpub/ckac094.066

**Published:** 2022-08-29

**Authors:** Susanne Andermo, Susanna Olin, Mai-Lis Hellénius, Anja Nordenfelt, Matthias Lidin, Gisela Nyberg

**Affiliations:** 1 Department of Global Public Health, Karolinska Institutet, Stockholm, Sweden; 2 Department of Neurobiology, Care Sciences and Society, Karolinska Institutet, Stockholm, Sweden; 3 Centre for Psychiatry Research, STAD, Karolinska Institutet and Stockholm County Council, Stockholm, Sweden; 4 Department of Medicine, Karolinska Institutet, Stockholm, Sweden; 5 The Foundation A Healthy Generation, The Foundation A Healthy Generation, Stockholm, Sweden; 6 Theme Heart and Vessels, Karolinska University Hospital, Stockholm, Sweden; 7 Department of Sport Science, The Swedish School of Sport and Health Sciences, Stockholm, Sweden

**Keywords:** Physical activity, Qualitative, Leaders, Families, Experiences

## Abstract

**Background:**

Family-based interventions may be a promising solution to increase children's physical activity, but there is a lack of knowledge on how to facilitate such interventions, specifically in socioeconomically disadvantaged areas. The aim of this study was to explore participants' and leader's experiences of the content and delivery of the family-based programme A Healthy Generation.

**Method:**

A Healthy Generation is a health-promoting programme, for families with children in grade 2 (8-9 years) including siblings. Intervention components are: activity sessions, parental support groups, healthy meals and health information. The programme is delivered twice a week for one school year in collaboration with local municipalities, health coordinators and a variation of invited sport organisations in socioeconomically disadvantaged areas. Data was collected through participant observations during activity sessions, interviews with leaders (n = 11), and four focus groups with parents (n = 27) who had participated in the programme. Data was transcribed verbatim and analysed using content analysis.

**Results:**

Leaders' and participants' experienced the programme to have an appealing ?Family-concept for joy, activity and integration?. The variation of activities provided opportunities and challenges to new interests, and the family approach were perceived as valuable for parental engagement and integration. To be ?A suitable programme for all participants?, activities directed to whole families needed to be simple and fun to keep all participants engaged, but also provide a progression for learning. Free and locally situated activities, meals and equipment for whole families facilitated participation, whereas lack of time and socio-cultural differences were barriers. The programme delivery consisted of ?A fruitful leadership collaboration? where health coordinators played an important role as coordinators of a heterogeneous group, so the invited leaders could focus on the content of their sport. They also provided participants with continuity and important reminders for participation during and in-between activity sessions.

**Conclusions:**

Participants' and leaders' experiences of a family-based health promoting programme give insight to the importance of local involvement, collaborative leadership and a well-adjusted family programme for health promotion. The study also draws attention to opportunities and barriers for increased integration through health promotion aimed at families in socioeconomically disadvantaged areas.

